# Current status, challenges and the way forward for dairy goat production in Europe

**DOI:** 10.5713/ajas.19.0327

**Published:** 2019-07-18

**Authors:** Francisco de Asís Ruiz Morales, José María Castel Genís, Yolanda Mena Guerrero

**Affiliations:** 1 Regional Director Western Europe of International Goat Association (IGA), Granada, 18193, Spain; 2 Economy Department, Andalusian Agricultural Research and Training Institute (IFAPA), Granada, 18004, Spain; 3 Agroforestry Science Department, School of Agricultural Engineering, University of Seville, Seville, Spain

**Keywords:** Dairy Goat, Local Breeds, Profitability, Cheese, Multifunctionality, Diversification

## Abstract

The aim of this review is to show the evolution of the dairy goat sector in Europe from all perspectives. Starting from the current situation, the challenges and future potential of this livestock system are presented, as well as strategies to overcome the difficulties faced. Europe holds 1.9% of the world goat population and produces 15.1% of goat milk recorded worldwide. The goat species plays a fundamental economic, social and environmental role in many regions of Europe. The wide diversity of production systems and autochthonous breeds makes the sector very heterogeneous. In order to improve viability, a number of strategies need to be adopted to solve the current problems such as a low profitability, absence of generational change and a little or no recognition of the social and environmental role of the sector. Some strategies to improve the situation of the European goat sector include: i) generating market value that will recognise the diversity of the dairy goat sector (breeds, feeding models, derived products…); ii) promoting and raising awareness of the functional attributes of goat milk and derived products so as to increase consumption; iii) assigning an economic value to environmental and social functions; iv) improving working conditions through technological innovation to make goat farming more attractive to young people; and v) processing more milk into cheese or other dairy products in production areas.

## HISTORY OF THE GOAT IN EUROPE

Following its domestication in 8000 BC and subsequent expansion across the Mediterranean, the goat has played an essential role in different eras and empires in Europe. In the late Greco-Roman world, goat breeding fulfilled important functions [1]. The vast and united Roman Empire facilitated the development of the goat sector, which survived into later centuries mainly in the eastern Roman Empire (Byzantium). The value of the goat in the survival of the desolate masses of Western Europe during the Middle Ages in periods of great famine is also worth recording [1].

In the muslim kingdom of Al Andalus (Spain), the goat provided different products besides milk and cheese, testimonies of which are found in the archaelogical remains and treaties of agriculture and gastronomy that are conserved from those times [2]. But not only in the Mediterranean, Norwegian dairy goat farming also has long-standing traditions, going back at least to the times of the Vikings, who may have taken goats from Norway to Iceland [3]. The domestic goat was taken to America from Europe by the Spanish settlers. During Christopher Columbus’ second voyage, goats were transported to the island of La Española, now Haiti and Dominican Republic, and later transferred to other islands and Mexico [4].

In the 19th century, a change in social structure and production methods was observed in Europe. Specialised production replaced traditional plant and animal farming and intensive production was designed to feed the urban populations that developed around fast-growing cities. Milk-producing cow units were set up and goats were relegated to marginal and poorer areas, where their role was limited to providing rural communities with milk, meat and manure for fertiliser [5]. Considered as marginal animals for subsistence of the poor, goats were often seen as harmful for forests and grazing and they were banned in many regions. Meanwhile in developed countries, beginning with France, growing interest in its dairy potential led to the creation of a dairy goat sector with a specific organisation for selection, processing and commercialisation [6].

In the 1990s, due to the European legislation on quality and hygiene standards laid down for milk and milk products ([EU] directives 92/46 and 94/71), the goat sector initiated an intense mechanization process, especially in the milking systems, which has greatly improved the health status of the herds and milk quality. This was accompanied by intensification of management systems and led to a decrease in the number of grazing farms [7].

Today goats play an important socioeconomic role in different regions of Europe, particularly the hills and mountains, and remote, marginal and even semi-arid areas [1,6]. Goats can adapt to different farming systems, climatic conditions and terrains, where they can take advantage of low-quality resources and transform them into high-quality products.

Goats also play an essential cultural role in festivities and celebrations such as the “Kukeri” in Bulgaria or the “Capra” in Rumania, besides other traditional popular customs.

In fact, dairy goats today carry out many different functions across the European territory [8]:

- a primary function at farm level producing milk and meat products
- a secondary function at industry or supply chain level processing dairy and meat products
- many tertiary functions: socio-cultural impact for the rural community, maintaining land equilibrium, landscape aesthetics, nutritional value, food security, hunting, tourism, fire protection etc.

This multifunctionality is very important in these less favoured and remote European areas where small ruminants are often the last possible economic activity.

## GOAT POPULATIONS, PRODUCTION AND TRENDS

Europe holds only 1.9% of the world goat population and unlike in other parts of the world, the European goat sector is linked to milk production and industrial cheese manufacture (Table 1). Despite having such a small goat population, Europe produces 15.1% of the world’s goat milk and 35.1% of the world’s goat cheese [9,10]. In the dairy sector of the European Union, goats provide only 1.5% of the total volume of milk produced, almost the same as sheep milk (1.8%), far behind cow milk (96.7%) [9].

The highest-ranking countries for goat populations as indicated in the livestock censuses in the EU are: Greece, Spain, Rumania and France (6.3, 3.0, 1.5, and 1.2 million head, respectively) [9]. In general, the EU goat farms are far more devoted to dairy products than in developing countries, especially in France, Greece and Spain, where annual goat milk production is 590, 562, and 491 million liters, respectively, which constitutes 76% of the total goat milk produced in the EU. France and especially Greece have increased their production in the period 2007 through 2017 by 2.8% and 12.4% respectively while Spain has maintained its level of production (0.5%). Other goat milk producing countries such as Holland have come into the scene with an increase in production of 35.5% in the last 10 years [9].

Most European goat milk is transformed into cheese in large dairy industries, which coexist with small local industries and artisanal farm dairies. These industries make pure goat milk cheeses, mainly in France, or blended cheeses with cow and/or sheep milk in Spain and Greece. Besides the demand for cheese, an increase has been observed in the demand for pasteurised goat milk for direct consumption and powdered goat milk for infant formulas. The organic dairy sector is growing in the European market and goat producers are increasing their presence with products such as cheese and different types of milk and yoghourt [11].

Many scientists focused on the functional properties of the goat milk [12] which include not only high nutritional value but also therapeutic value and dietary characteristics [13]. On the other hand, goat farming is gaining recognition for its environmental and social role, particularly in areas that offer very little potential for crop production and where goat farming is associated with use of pastures and natural resources [14]. Society in Europe is increasingly concerned about environmental issues and sees this type of farming system as a tool to support land management. Actions are being taken to assign an economic value to this role, such as paying the farmer to reduce biomass fuel through grazing and thus contribute to wildfire prevention [15,16].

The abandonment of rural areas is a common problem faced all over Europe, therefore anchoring the population is an objective pursued by the common agricultural policy and goat farming and all aspects related to this subsector have an essential role to play and are to be reinforced as a priority issue [14].

## DAIRY GOAT PRODUCTION SYSTEMS IN EUROPE

Europe is the continent with the widest caprine biodiversity, with 187 goat breeds making up 33% of the goat breeds recognised worldwide [17]. Diversity of ecosystems throughout Europe has given rise to this heterogeneity of goat breeds, which is a strength of this livestock sub-sector. Maintaining the biodiversity of animal breeds is an important condition for animal production to adapt to the changing conditions of breeding and production systems in the future [6].

In this context, there are breeds with large population sizes, characterised by a large milk production leading to exports to other countries that no longer rear their own autochthonous breeds, as is the case with Saanen and French Alpine, or the Murciano-Granadina in Spain. On the other hand, there are breeds in the opposite position, with small population sizes and no conservation schemes or breeding programmes because of their remoteness or low productivity. They are often in a critical situation and on the brink of extinction [6]. The Table 2 includes the production data of different European goat breeds.

The high production of the French and Spanish breeds mentioned earlier is linked to their breeding programmes and to other management factors such as feeding, health care, facilities and equipment, etc., that cannot be implemented in many European countries.

In this context of genotype diversity, traditional systems of meat–milk production co-exist with intensive milk production systems. There is a wide range of dairy goat farms, divided into two types of systems; those in which goats are kept permanently indoors and those where goats are on pasture with different grazing times [22].

**Figure 1:**
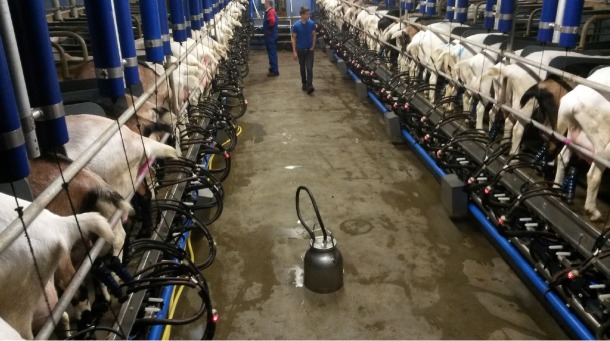
Saanen and Alpine goats in milking parlour.

Use of confined systems has increased in recent years for different reasons such as entry into force of mandatory regulations, increase in demand for animal products, lack of available pastures, easier animal husbandry and, sometimes more profitability per animal [7]. These systems are characterised by the use of high-yielding breeds such as French Saanen and Alpine or the Spanish Murciano-Granadina, Malagueña and Florida. France, Holland and many areas of Spain use confined systems. Animals are fed on concentrates and forages provided directly in the feeding trough. Production is less seasonal than in the grazing systems and can meet the demand from the big dairy industries that pay more to farmers that can ensure a constant milk supply all year round. This model is highly mechanized, with automatic milking systems, mechanised feed distribution, batch kidding to ensure milk supply throughout the whole year, artificial insemination, etc (Figure 1). The Table 3 includes technical indicators for confined goat farms in three countries of the EU.

Grazing systems are the most common farming practice in the countries of southeastern Europe and in the mountainous, semi-arid and marginal areas of traditional goat farming countries such as France, Spain and Italy. It is difficult to characterise this production model precisely as it includes a wide range of management systems namely: continuous grazing, seasonal grazing, transhumance, use of natural and/or cultivated pastures, etc. [25–27]. This diversification can be explained by a number of environmental factors such as the climate, soil and vegetation conditions that vary enormously between regions, as well as noteworthy social factors such as advisory services from goat breeders associations, land availability or recognition from society of the professional importance of the goat herder [6,14,22].

The grazing systems in France are mainly located in the South East. The French grazing systems are differentiated from each other according to production purpose as they either produce milk to be sold to the dairy industry or they make their own farmhouse cheeses (*fermiers*) [18,25]. The grazing farms are medium-sized, sometimes rearing other ruminant species, consume less concentrates and forage than the intensive systems and consequently produce less milk per goat. The farmers that make and sell cheese on the farm have much smaller herds and consume more forage. Of all the European grazing models, the French farms produce the most milk but also use the most feed inputs (Table 4).

**Figure 2:**
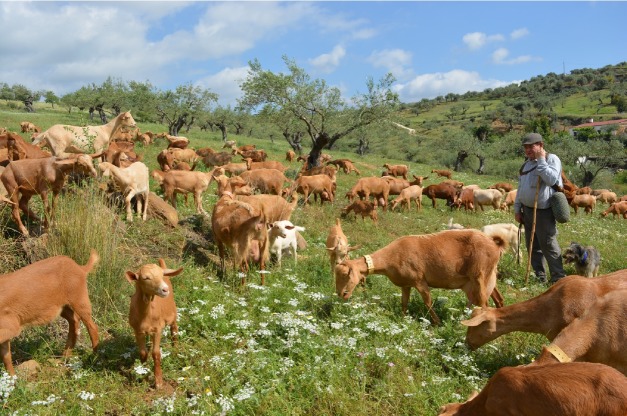
Herd of Malagueña goat breed in pastoral systems (Source: CABRAMA).

In Spain, grazing goat systems have undergone a similar intensification and mechanization process to systems rearing other livestock species. The main changes have been milking mechanisation, use of milk cooling tanks, more use of concentrates and reproduction management [7]. Spanish grazing goat farms are located mainly in mountain areas of the South and South East (Figure 2), and in the semi-arid areas of the East. Herds are medium-sized with less milk production than the French grazing systems and use less forage [25,28]. Milk production is destined mainly to the dairy industry and very little is used to make farmhouse cheeses, following the French model, or to make artisan cheeses [22].

Traditional goat grazing systems in Greece are less mechanized than in France and Spain; they have few milking parlours, use a minimum amount of commercial concentrates and do not have structural facilities such as livestock handling chutes. As herds are medium-sized and use few feed inputs, farms are more self-sufficient but they also produce less milk [27].

In the other eastern European countries, with the exception of Greece, dairy goat herds are small. The census of some local breeds is a growing cause for concern as they are being replaced by more productive foreign breeds such as Saanen and Alpine. All farms are managed extensively with limited feed supplementation in some cases. Goats are still milked by hand in most countries, although portable milking machines are starting to be used in Croatia and Hungary, for example, and only Slovenia has installed milking parlours [29].

Another characteristic of goat farms, irrespective of the farming system, is their family structure. Farms are family-run, the work force is made up mainly of family members and hardly any outside labour is hired [14,21,30].

Generally in the EU, goat farms that are oriented towards the production and sale of raw milk face profitability problems due to increasing operations costs and lower sales prices for milk and meat products. The economic performance of dairy farms is better than meat farms which would not be able to survive without the subsidies from the European Union [14].

The Table 5 includes the economic results of French farms with different feeding models. It is very complicated to make comparisons of goat farming profitability in different countries as there is practically no data available (except in France where the Institut de l’Elevage has led data collection for a number of years and has published annual economic data for all the French production models). In this case, the systems that use silage and alfalfa hay are reported to be more economically viable.

Goat farming systems in Europe are much less profitable than other economic activities and face other challenges such as an enormous administrative burden, harsh working conditions and lack of services in rural areas. This makes them very vulnerable and an increasing number of farmers abandon farming, irrespective of the size of the farm [14].

## DAIRY GOAT PRODUCTS IN EUROPE

France is the driving force behind the European goat sector in the large-scale dairy industry. Countries like Spain and Holland sell part of their production to France which imports around 99 million litres of goat milk a year for cheese manufacture [31].

In Spain, goat milk that is not exported is used to make blended cheeses (cow-sheep-goat) and to a lesser degree pure goat cheeses. Most goat milk is processed by large industries located far away from the production areas. In Holland, besides selling milk to France, farmers export powdered goat milk to the Asian market for infant formulas. In Greece, only 21% of goat milk is sold to dairy industries. The rest is used for on-farm cheese production and mostly sold directly to consumers [32].

Farmhouse and artisan cheese is one of the sales destinations of goat milk in the EU that is worth encouraging, as the farmer benefits from the added value of the processing and sale, making the farm more profitable. With this sales model, farms either sell their milk to a local artisan cheesemaker or make their own farmhouse cheeses. Volumes are small in both cases and the milk is processed in adapted facilities. In France 20% of goat milk is processed according to this model [18]; percentages in Greece and other countries of Eastern Europe are estimated to be higher. Both models are also found in Spain, although there are differences between regions. In Andalusia (south Spain and the second goat milk producing region of the EU), only 5% of milk produced is processed into artisan cheeses [33]. In the Canary Islands there is much more artisan cheese production, with about 380 artisan cheese plants or on-farm facilities [34]. The adaptation of the EU regulation in Europe has enabled this type of cheesemaking to develop.

The criterion followed by the cheese-making industries when paying for raw goat milk is based on a number of parameters such as milk fat and protein content, absence of inhibitors, presence of germs, somatic cell concentration and seasonality of production - lower prices in spring and higher prices in autumn-winter [35]. This means that there may be large fluctuations in the final milk price throughout the year (between 0.6 and 0.9 € per litre).

Official data reveals that around 201.29 thousand tonnes of pure goat cheese is produced in the European Union. France is the main producer (52%), followed by Spain (25%), Holland (13%), and Italy (3%). There is no official data for Greece [9].

Goat’s milk has been a traditional food of southern Europe. The production of dairy products from goat milk such as cheese is considered part of its ethnological, gastronomic and cultural heritage. The European Union adopted a regulation to protect these products through quality labels linked to the territory (Council Regulation [EEC] no. 2081/92 [1992]). The most restrictive is the protected designation of origin (PDO), where the whole production process must take place in the territory itself and must be linked to other characteristics such as autochthonous breed, feed or traditional cheese-making recipe. According to quality products register from European Union (EU-27), there are currently 23 pure goat cheeses, 23 goat and sheep blended cheeses, 7 goat and cow blended cheeses, and 7 goat, sheep and cow blended cheeses protected under this quality scheme.

According to statistics, pure goat cheese consumption has risen in recent years. French households increased purchases of goat cheese by 12.0% between 2013 and 2017 with a volume of 57,030 t; in 2017 the average retail price for goat cheese in France was 12.3 €/kg [18]. In Spain household consumption of goat cheese also increased in more or less the same period (2014–2017) by 7.6%, at an average retail price of 10.9 €/kg.

Finally, different European research groups have reported positive results for goat milk and dairy products in comparison with cow and sheep milk, highlighting its functional properties and positive effects for human health [13].

## ENVIRONMENT AND THE GOAT SECTOR

The term ecosystem services have been introduced in recent decades. This concept refers to “the direct or indirect contributions of ecosystems to human welfare” [36] and has acquired great importance in the analysis of agricultural and livestock systems. Ecosystem services are classified in three categories: provisioning, regulating and cultural. Provisioning services are provided directly by the ecosystems (e.g. food, freshwater, wood, cellulose, genetic resources). Regulating services refer to indirect contributions made from the ecosystems’ processes (e.g. climate regulation, flood control, water purification). Cultural services are immaterial, intangible contributions made by the ecosystems in the form of personal experiences (e.g. spiritual, recreational, touristic, aesthetic, educational, feeling of identity, cultural inheritance).

**Figure 3:**
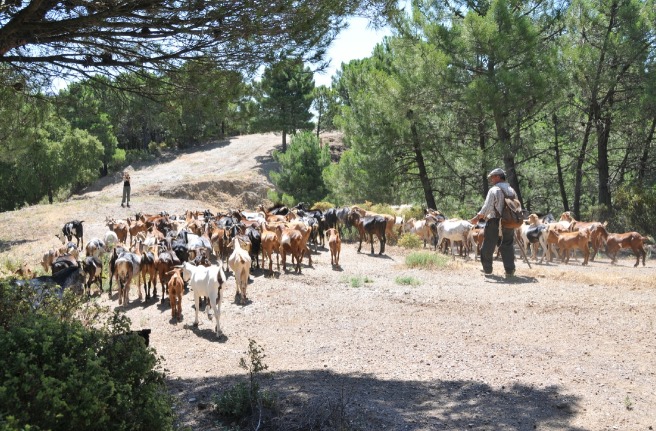
Sheperd wih dairy goat herd in fuelbreaks for wildfire prevention (Source: RAPCA).

The role played by goat farming in relation to the ecosystem services it provides is well known, namely seed dispersal of Mediterranean species through endozoochory, litter decomposition and balance between authochthonous and invasive species, and land conservation [37]. The presence of goats helps to manage the land, shape the landscape and reduce biomass fuel (Figure 3), among other benefits [15,16,37]. Furthermore, taking goats to pasture rather than keeping them indoors means that much less non-renewable energy is used, for example in the production and transport of concentrates [38] and less net greenhouse gas emissions are produced due to increased carbon sequestration [39], one of the main challenges addressed in the EU Horizon 2020 programme. Moreover, well managed goats are perceived by society to have a strong, positive, naturalist, and environmentally-friendly image [40].

From the sociocultural point of view, dairy goat farming helps to maintain cultural and ethnological traditions and typical products. It also contributes to anchoring the rural population in disadvantaged areas as there are goat farms in 70% of geographically marginal areas, including isolated, remote regions of difficult access [14].

Despite agri-environment payments provided by the EU agricultural policy, this range of services is not recognised, and the farmer only receives income from milk and meat sales (provisioning services). In order to contribute to the economic sustainability of goat farming, not only should the environmental and social role be recognised, but it should also be paid for, thus diversifying and increasing the income received by farmers. Timid attempts are being made in this direction in Europe, to generate commercial value for the positive externalities of traditional goat farming systems and to encourage ways to make goat farming more sustainable in environmental and social terms [33,40]. People are talking along these lines in the European dairy livestock farming sector in general and in the goat farming sector in particular, addressing concepts such as organic, agroecological, animal welfare, biodiversity, GMO free etc.

Organic production in the European dairy sector is growing considerably. Based on the EU Regulation (EU) 2018/848, organic goat milk production increased by 47.2% in the period 2012 through 2017. The total number of goats in organic production (meat and milk) is 833,087 heads, producing 49.4 million litres of milk [41]. Organic production brings with it an increase in milk prices and responds to the needs of those consumers that are more conscious about their health and the environment [18]. Goat farming is still very diversified, with numerous autochthonous breeds that are well adapted to a range of pasture-based ecosystems. These systems can adapt their management practices to the rules of organic production and achieve extra quality without making big operational changes [42]. Therefore in forthcoming years this production model will continue to increase in response to the demand of European consumers.

Another important dimension of the added-value generated by PDO cheeses is that they can serve as a benchmark to measure biodiversity conservation. Studies are unanimous in their description and analysis of the PDO scheme as a tool to enhance the conservation of biological diversity such as local breeds, grass species for pastures, cheese-making processes or organoleptic quality and diversity.

Another example of generating value through livestock ecosystem services lies in the use of grazing livestock systems to prevent wildfires. In European countries such as France and Spain, shepherds and goat herders are paid directly to use their animals to control biomass fuel in inaccessible areas, as it is cheaper than using traditional mechanical techniques to remove scrub and undergowth and is positively valued by society [16,43].

## DIAGNOSIS AND STRATEGIES TO IMPROVE THE EUROPEAN DAIRY GOAT SECTOR

Different sources of information have been used to make a diagnosis of the current situation of dairy goat farming in Europe through a SWOT analysis of the internal characteristics (Weaknesses and Strengths) and external situation (Threats and Opportunities) with a view to implement improvement strategies [14,21,22,25,32]. The strengths determined by the internal analysis are: i) goats’ adaptation to different environments with remarkable production levels; ii) diversity of breeds and production; iii) better functional quality of goat milk in comparison to cow and sheep milk; iv) high organoleptic and nutritional quality of derived goat products; v) family-run and mainly located in marginal areas and vi) increased organisational capacity and ability to structure the sector, with potential for improvement. The weaknesses include: i) autochthonous breeds in danger of extinction; ii) lack of profitability in comparison to other economic activities; iii) harsh working conditions, especially on the pasture-based dairy goat farms; iv) a need for training and for public advisory services in some countries; v) deficient structure of the sector preventing commercial development; and vi) scarce representativity of the sector in the decision-making bodies (lack of information and political power and low economic importance).

The opportunities detected in the external analysis are: i) an increment of EU aid as a way to pay for the ecosystem services provided by this livestock farming located in rural areas; ii) increased demand and consumption of dairy goat products in general and in particular for infant formulas; iii) the positive image that consumers have of goat dairy products as high quality, sustainable and natural; iv) the diversity of dairy products made from goat milk; v) new technologies that can be applied to the livestock sector, facilitating management and making goat farming more attractive to young people; and vi) the increasing importance conferred to the environmental and social role of grazing livestock systems, which can become another source of income for the farm. The threats identified in the analysis are: i) a cattle-dominated dairy sector in Europe with lower milk prices than for goat milk; ii) insufficient government investment in research and experimentation of the goat sector, therefore insufficient progress in improving the sustainability of this subsector; iii) increase in production costs (energy, feed, medicines) that may not be accompanied by an increase in the price of the products leading to a loss in profitability; iv) a milk market dominated by an oligopoly of industries, generating a lack of balance between the producer and the processor; v) more consumers are reluctant to buy animal products; and vi) lack of generational replacement in goat farming.

Based on this analysis, some strategies have been drawn up to improve and optimise the European dairy goat sector: i) to continue to work on product diversification, not just from the technological side, but also to take into account the biodiversity of the breeds, use of natural pastures and feeding systems (organic, grazing, transhumanc, etc.) or quality labels (PDO, protected geographical indication, mountain product, etc.), and innovating in new dairy products; ii) to promote and publicise the functional attributes of goat milk and its derived products to increase consumption; iii) to encourage farmers to make dairy products in the milk producing areas, either following the farmhouse cheese or “fermier” model or through cooperatives or other forms of association to process milk for their members; iv) to create working groups and projects at European level to monitor the real situation of the goat sector through associations in each country, research centres and international associations such as the International Goat Association; v) to encourage uptake of new technologies through comprehensive management programmes, tracking and monitoring systems for grazing farms, surveillance cameras, techniques to improve reproduction efficiency; vi) to anchor the rural population through direct aids paid for production or to incorporate young farmers, as well as other support linked to daily family life (schools, healthcare, leisure, etc.); and vii) to establish tax breaks for economic activities in these areas.

## CONCLUSION

The goat has been present in Europe since ancient times. Today there is a wide diversity of breeds and production systems. It is a sector which is highly specialised in milk production and pure or blended goat cheeses. Even though dairy goat production is not a livestock subsector that stands out for its economic importance, it is of considerable importance in environmental and social terms, making it a strategic sector that should be maintained and improved.

Although European goat farming has become technified and intensified in recent years, there are still many areas where goats have close links with the territory, generating important ecosystemic services.

Improvements still have to be made to aspects that can make this activity profitable and attractive to young people, to raise awareness among the general public of the value of goat products and obtain appropriate prices for them and likewise recognise and value their contribution to society and to the environment, especially in the more disadvantaged rural areas.

## Figures and Tables

**Table 1:** Dairy goat population and milk production in the World, the European Union and the main European dairy goat countries

Items	Population (no. of head)	Milk production (tonnes)
World	1,034,406,504	18,656,727
Europe	19,290,067	2,824,715
European Union-27	15,157,721	2,150,039
Greece	6,300,000	562,491
Spain	3,059,731	491,374
Rumania	1,483,100	-
France	1,223,816	590,000
Holland	532,870	246,562

Source [9].

**Table 2:** Milk production and composition of different European breeds

Breed	Country	Production per lactation (kg)	Days of lactation	Fat (%)	Protein (%)
Alpine	France	789	250	3.72	3.29
Saanen	France	784	250	3.57	3.18
Murciano-Granadina	Spain	583	285	5.60	3.60
Malagueña	Spain	502	268	4.80	3.20
Payoya	Spain	440	219	4.20	3.50
Sarda	Italy	173	-	4.57	4.35
Carpathian	Rumania	240–280	270	4.50–5.00	-
Balkan	Serbia	281.3	234	3.85	3.51

Source [18–21].

**Table 3:** Production indicators of confined goat farms in France, Spain, and Holland

Items	France	Spain	Holland
Year	2016	2015	2015
Number of heads	516	344	850–900
Kg concentrates per goat and year	350	555	-
Kg forage per goat and year	678	257	-
Litres produced per goat and year	918	493	950–1,000

Source [18,23,24].

**Table 4:** Production indicators of grazing dairy goat farms in different countries of Europe

Items	France (milk)	France (farmhouse cheese)	Spain	Greece
Year	2016	2016	2011	2011–2012
No. goats per farm	245	100	372	254
Kg milk per goat and year	674	796	295*	172
Kg concentrates per goat and year	340	311	337	180
Kg forage per goat and year	486	789	65	108

Source [18,27,28];

* milk sold.

**Table 5:** Economic indicators of goat farms in France according to feeding system

Items	Grazing	Fresh forage	Grass silage	Corn silage	Alfalfa hay
No. goats per farm	257	546	402	453	316
Kg milk per goat and year	705	860	830	931	925
Price of milk (€ per l)	0.682	0.718	0.682	0.702	0.702
Milk revenue (€ per goat and year)	480.8	617.5	566.1	653.6	649.4
Feeding cost (€ per goat and year)	152.9	248.5	179.3	220.6	245.1
Gross margin (€ per goat and year)	364	364	427	456	423

Source [18].
